# Assessing Ambiguity of Context Data in Intelligent Environments: Towards a More Reliable Context Managing System

**DOI:** 10.3390/s120404934

**Published:** 2012-04-17

**Authors:** Aitor Almeida, Diego López-de-Ipiña

**Affiliations:** Deusto Institute of Technology (DeustoTech), University of Deusto, 48007 Bilbao, Spain; E-Mail: dipina@deusto.es

**Keywords:** fuzzy logic, uncertainty, vagueness, context-aware, data fusion, inference

## Abstract

Modeling and managing correctly the user context in Smart Environments is important to achieve robust and reliable systems. When modeling reality we must take into account its ambiguous nature. Considering the uncertainty and vagueness in context data information it is possible to attain a more precise picture of the environment, thus leading to a more accurate inference process. To achieve these goals we present an ontology that models the ambiguity in intelligent environments and a data fusion and inference process that takes advantage of that extra information to provide better results. Our system can assess the certainty of the captured measurements, discarding the unreliable ones and combining the rest into a unified vision of the current user context. It also models the vagueness of the system, combining it with the uncertainty to obtain a richer inference process.

## 1. Introduction

Intelligent environments host a diverse and dynamic ecosystem of devices, sensors, actuators and users. When modeling real environments certainty cannot be taken for granted. Reality, and hence the user context [1], is ambiguous. Sensors and devices are not perfect and their measurements carry a degree of uncertainty; for example, several thermometers in the same room can provide conflicting temperature values and there always exists the human factor. Not every user can provide the exact temperature they want for their bath—most of them will only say that they want it “warm”. For this reason, when developing smart spaces and ambient intelligence applications, it is important to address ambiguity in order to model the context more realistically. To provide our systems with this feature, we have centered our work on two aspects of ambiguity: uncertainty and vagueness. We use uncertainty to model the truthfulness of the different context data by assigning to them a certainty factor (CF). This way we can know the reliability of each piece of information and act accordingly. This knowledge also allows us to create a more robust data fusion process to resolve the problem of the existence of multiple providers for the same piece of information at the same location. On the other hand, vagueness helps us to model those situations where the boundaries between categories are not clearly defined. This usually occurs when users are involved. Different users will have different perceptions about what constitutes a cold room or a noisy environment. We have addressed this problem using fuzzy sets to model the vagueness.

By taking into account the ambiguity in the context information our aim is to improve the reliability of context management systems. As Black argues, vagueness should not be equated with subjectivity [2]. From our point of view (as we will discuss in Section 5) modeling uncertainty and vagueness improves the precision of the system. With this information the system is able to better assess the actual state of the context, being able to react to a broader range of situations. In this paper we will describe the three main components of the ambiguity conscious framework we have developed. First we will describe the ontology created to model the uncertainty and vagueness in context. Then we will discuss the data fusion process that takes place to infer the real status of the locations using multiple measures. Finally we will describe the implemented inference mechanism that processes ambiguity as a whole, combining vagueness and uncertainty. The outline of the paper will be the following: in Section 2 we will analyze the related work, in Section 3 we will describe the created ontology, in Section 4 we will explain how the framework works and the inference that takes place within it, in Section 5 we will describe three user cases to illustrate the necessity of taking into account the ambiguity of the context data. Finally, in Section 6 we will discuss our conclusions and the next steps we intend to take.

## 2. Related Work

As discussed in [3], modeling the context with ontologies offers the following advantages:

- Ontologies are the most expressive approach to model context.
- Composition and management of the model can be done in a distributed manner.
- It is possible to partially validate the contextual knowledge.
- One of the main strengths of ontologies is the simplicity to enact the normalization and formality of the model.

Multiple ontology based context models have been developed. SOUPA [4] is a set of ontologies oriented to ubiquitous and pervasive applications used by the COBRA project [5]. It is composed by two sub-sets: SOUPA Core and SOUPA Extensions. The SOUPA Core defines the elements that are present in any ubiquitous application, while SOUPA Extensions support more specific applications. CONON [6] is used by the SOCAM [7] project to model the context of pervasive computing applications. It is also divided into two sets, one with the general information shared between all the applications and the other one domain specific. CODONT [8] is used by the CODAMOS project and its main aim is to create an adaptable and flexible infrastructure for AmI applications. In [9] Hervás *et al.* present a formalized context model for offering visualization services to the users depending of their situation, needs and preferences. The proposed model is based on four ontologies: users, devices, environment and services, describing concepts of intelligent environments and their relationships

Several authors have worked on combining indetermination or vagueness with ontologies. An extensive survey can be found in [10]. In the case of indetermination, in [11] its authors present a probabilistic generalization of OWL called PR-OWL, based in Multi Entity Bayesian Networks (MEBNs) which allows the combination of first order logic with Bayesian logic. This ontology represents the knowledge as parameterized fragments of Bayesian networks. In [12] the authors propose another probabilistic generalization of OWL called BayesOWL which also uses Bayesian networks. The authors suggest a mechanism which can translate an OWL ontology to a Bayesian network, adding probabilistic restrictions when building the network. The created Bayesian network maintains the semantic information of the origin ontology and allows ontological reasoning modeled as Bayesian inference. The authors in [13] describe another integration of OWL with Bayesian networks, a system named OntoBayes. It uses an OWL extension annotated with probabilities and dependencies to represent the uncertainty of Bayesian networks. These probabilistic extensions are not confined to OWL only; in [14] an extension for OWL Lite is discussed and in [15] and [16] extensions for RDF are presented. Several authors have also addressed the combination of the vagueness (represented as the usage of fuzzy sets) with ontologies. In [17] authors analyze how SHOIN could be extended adding the possibility of using fuzzy sets (f-SHOIN). They also propose a fuzzy extension for OWL. In [18] authors describe a fuzzy extension for SROIQ(D) and present an Fuzzy OWL2 Ontology. In [19] a fuzzy ontology for the management of medical documents is discussed. This ontology can store different membership values. Additionally the author has created a mechanism based in the occurrence of keywords in the title, abstract or body of the document to calculate the membership value of the different categories. In [20] its authors describe a fuzzy ontology used to automatically create summaries of news articles. These authors have also created a mechanism for the automatic creation of the fuzzy ontology based on the analysis of the news. Finally, in [21] the authors propose a mechanism to create automatically fuzzy ontologies. The created ontologies include the membership values of the different terms.

Our system combines both approaches, enriching the context model with both uncertainty and vagueness. It also takes into account the interaction between them (as explained in Section 4), assessing how the vagueness modifies the resulting certainty factor. By assessing both aspects of ambiguity the resulting system is able to model a broader range of situations and interactions in smart environments.

## 3. AMBI^2^ONT: An Ontology for the Ambiguous Context

One of the problems we encountered in modeling context data in previous projects was the use of the uncertainty and vagueness of the gathered information. In the Smartlab project [22] none of this information was used, which led to a loss of important data like the certainty of the measures taken by the sensors. In the Imhotep framework (http://www.morelab.deusto.es/imhotep/) [23] we started using fuzzy terms to describe a small part of the context (the capabilities of mobile devices and users) in a human-friendly manner. Our objective with the work presented in this paper was to develop a framework capable of managing the ambiguity and incertitude that often characterizes the reality. To do this we have created an ontology that models these concepts.

**Figure 1.**
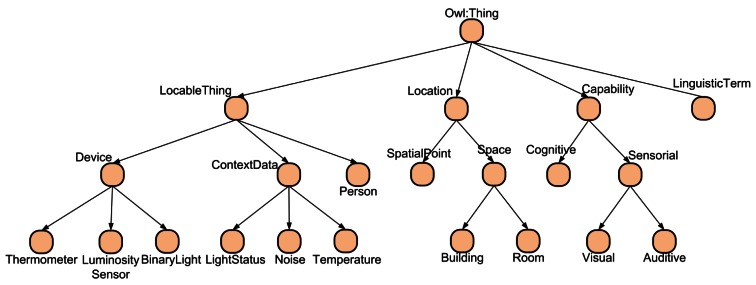
Subset of the main ontology concepts.

As shown in Figure 1 the main elements of the ontology are:

- *Location*: The subclasses of this class represent the location concepts of the context. In our system we have three types of locations: points, rooms and buildings.
- *LocableThing*: The subclasses of this class represent the elements of the system that have a physical location. It contains three subclasses: the Person subclass represents the users, the Device subclass models the different devices of the environment and the ContextData subclass models the measurements taken by the sensors. As we will explain in the next section, there are two types of measurements, those taken by the devices and the global measures for each room calculated by our data fusion mechanism. Figure 1 shows a subset of the type of context data taken into account in the ontology.
- *LinguisticTerm*: This class models the fuzzy linguistic terms of the values of the context data. The ontology only stores the linguistic term and membership value of each individual of context data. Currently the ontology does not model the membership functions and rules used by the inference engine.
- *Capability*: The subclasses of this class model the capabilities of users and their mobile devices. One objective of our framework is to be integrated with the Imhotep Framework that allows creating adaptive user interfaces that react to these capabilities and the changes on the context.

### 3.1. Modeling Uncertainty and Vagueness

**Figure 2.**
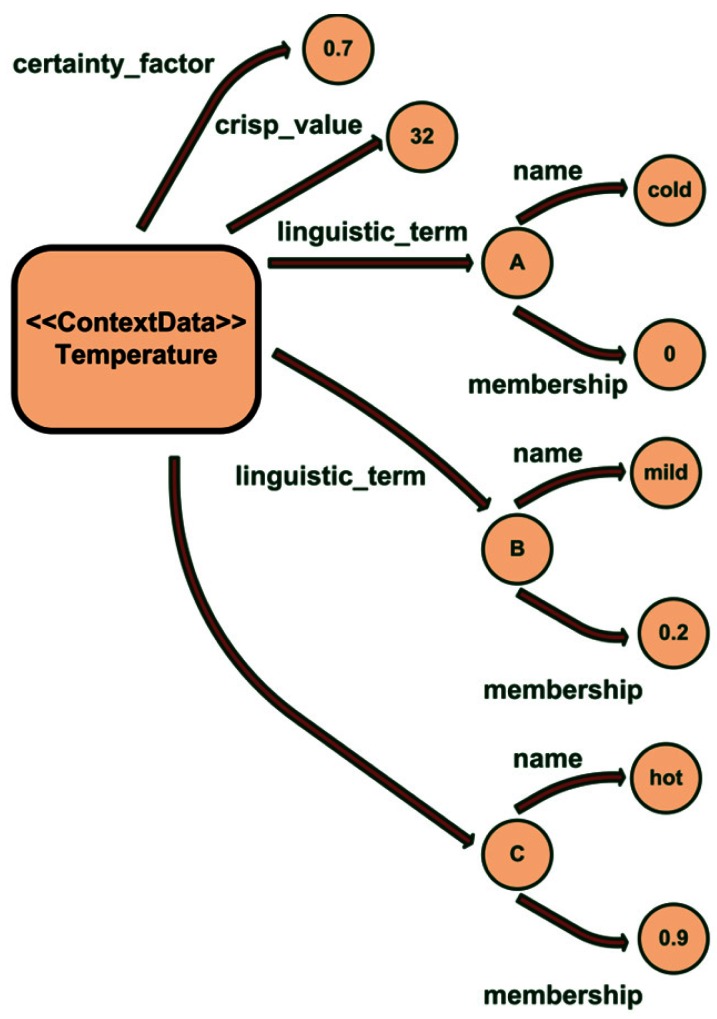
Example of the ambiguity data for a temperature measure stored in the ontology.

Our ontology models two aspects of the ambiguity of the context data: the uncertainty (represented by a certainty factor, CF) and the vagueness (represented by fuzzy sets). Uncertainty models the likeliness of a fact, for example “the temperature of the room is 27 °C with a certainty factor of 0.2 and 18 °C with a certainty factor of 0.8” means that the value of the temperature is more probably 18 °C (but it cannot be both of them). In the case of vagueness it represents the degree of membership to a fuzzy set. For example “the temperature of the room is cold with a membership of 0.7” means that the room is mostly cold. In Figure 2 it can be seen how those values are stored in the ontology. Each ContextData individual has the following properties:

- *crisp_value:* the measure taken by the associated sensor. In our system a sensor is defined as anything that provides context information.
- *certainty_factor:* the degree of credibility of the measure. This metric is given by the sensor that takes the measure and takes values between 0 and 1.
- *linguistic_term:* each measure has its fuzzy representation, represented as the linguistic term name and the membership degree for that term.

This can be seen in the example shown on Figure 2. The temperature measurement has a crisp value of 32 °C with a certainty factor of 0.7. After processing that crisp value with the associated membership functions our system has inferred that the membership degree for cold is 0, for mild is 0.2 and for hot is 0.9; so the room is mainly hot.

## 4. Semantic Context Management for Ambiguous Data

**Figure 3.**
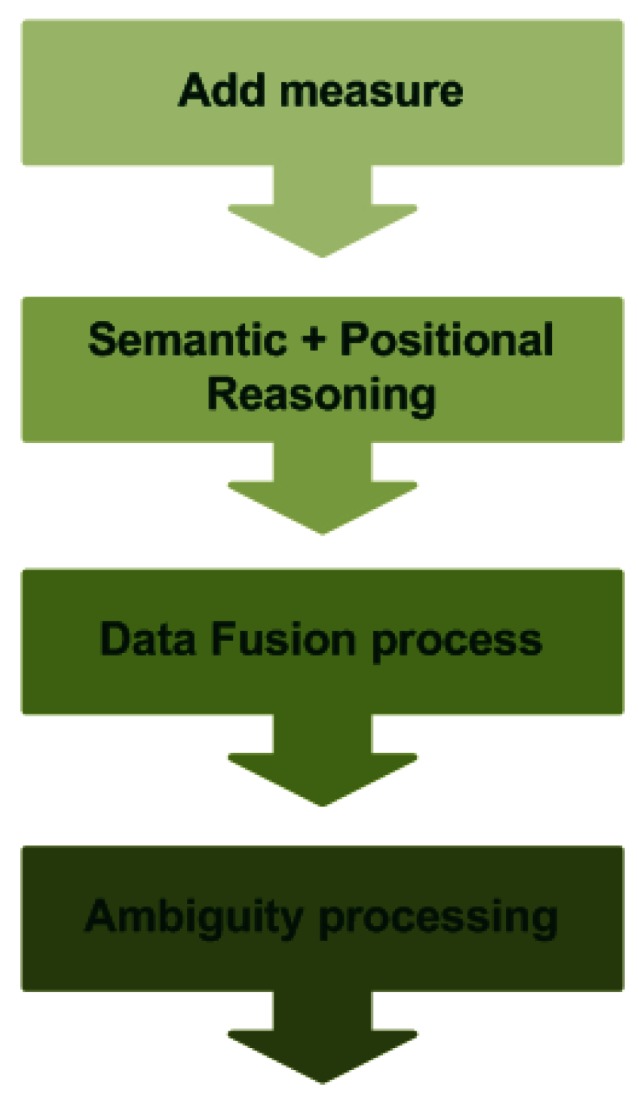
Context management process.

The semantic context management is done in four steps (see Figure 3): add the measures to the ontology, process the semantic and positional information, apply the data fusion mechanism and process the ambiguity contained in the data.

### 4.1. Adding the Measure

To add a measurement to the ontology the sensor must provide the measurement type, its value, location and a certainty factor. We assume that each sensor knows its certainty factor based on its type and manufacturer. We also assume that the certainty factor of the sensor can change over time depending on the environment (e.g., a thermometer can be pretty accurate for temperatures between −10 °C and 50 °C, but the measurement quality can degrade outside that range). For that reason the sensor certainty factor is not stored in the ontology when the sensor registers itself.

### 4.2. Processing the Semantic and Spatial Data

**Figure 4.**
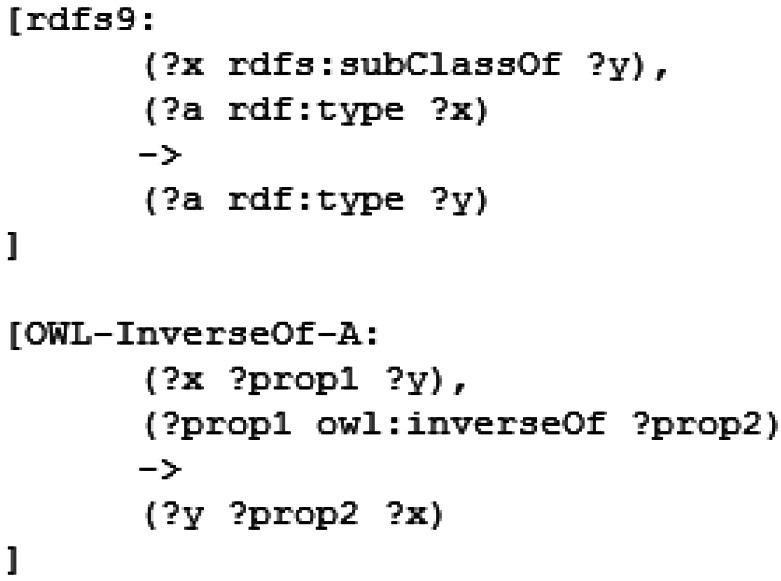
An example of the implemented semantic rules. The first rule is extracted from the RDF Model Theory and models the transitivity of the subClassOf relationship. The second rule is extracted from the OWL Model Theory and models the behavior of the inverse properties.

Once the measurements have been added, we apply a semantic inference process to achieve two goals: make explicit the hidden implicit knowledge in the ontology and infer the positional information of each measurement. To do this we use two different sets of rules: the semantic rules and the spatial heuristic rules. To make the semantic reasoning less cumbersome we implement a subset of the RDF Model Theory [24] and the OWL Model Theory [25]. An example of the used rules can be seen in Figure 4.

**Figure 5.**
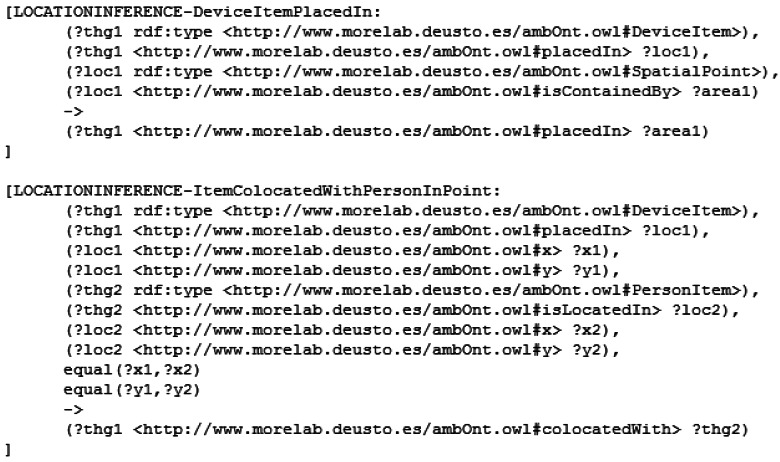
Example of the spatial inference that takes place in the system. The first rule is used to infer the area where the device is places in using the (x,y) coordinates of that device. The second rule infers if a device and a person are placed in the same place.

The spatial heuristic rules are used to infer higher level spatial information from the coordinates provided by the sensors. This information comprises data like the room in which the sensor is located; the devices, people and sensors surrounding it and the relative location to other LocableThing-s (refer to Section 3 for more information about the elements of the ontology). An example of the used rules can be seen in Figure 5. In the first rule a device's area of location is inferred using its (x,y) coordinates. The second rules checks if a device and a person are in the same position.

### 4.3. The Data Fusion Process

Once the location and semantic information of the measurements has been inferred and processed, the data fusion process is applied. From the previous step we can infer that each room can have multiple sensors that provide the same context data (e.g., various thermometers located in the same room). Usually the values and certainty factors of those measurements do not coincide. To be able to take the proper actions we need to process those differing measurements to assess the real status of the room. To tackle this problem we have created a data fusion mechanism that refines those individual measurements into a single global one for each room. We have implemented two types of strategies for this process: tourney and combination.

Using the tourney strategy the measure with the best CF is selected as the global measure of the room. On the other hand the combination strategy has three different behaviors, as stated in [26]:

- *Severe:* The worst certainty factor from all the input measurements is assigned to the combined measurement.
- *Indulgent:* The best certainty factor from all the input measurements is assigned to the combined measurement.
- *Cautious:* An average certainty factor is calculated using the certainty factor from the input measurements.

To determine the combined measurement value we weight the individual values using their certainty factors, as seen in the following equation:

(1) $$m_{\text{global}}=\frac{\sum_{i=0}^{n}(m_{i}\times cf_{i})}{\sum_{i=0}^{n}cf_{i}}$$

where:

- *m_i_*: the measurement values
- *cf_i_*: the measurement certainty factor

It is also possible to configure a minimum CF level for the measurements. Measurements with a CF value below this threshold will not be taken into account in the calculations. In Table 1 we show an example of how the data fusion process works when the combination strategy is used.

### 4.4. Processing the Ambiguity

As explained previously we model two aspects of the ambiguity: the uncertainty and the vagueness. To be able to reason over this information we have modified the JFuzzyLogic [27] Open Source fuzzy reasoner to also accept uncertainty information. JFuzzyLogic follows the Fuzzy Control Lenguage (FCL) [28] standard for its rule language.

**Figure 6.**
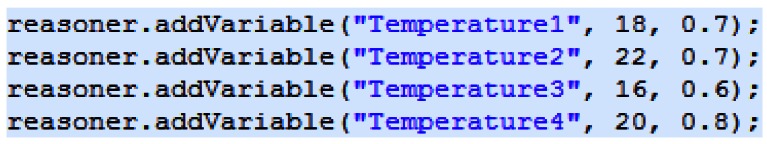
Use of the modified reasoner to process the data of the previous example.

The modified reasoner supports two types of uncertainty: uncertain data and uncertain rules. The first type occurs when the input data is not completely reliable (as seen in the example shown in Table 1). To support this type of uncertain data we have modified the API of the reasoner (see Figure 6).

**Figure 7.**
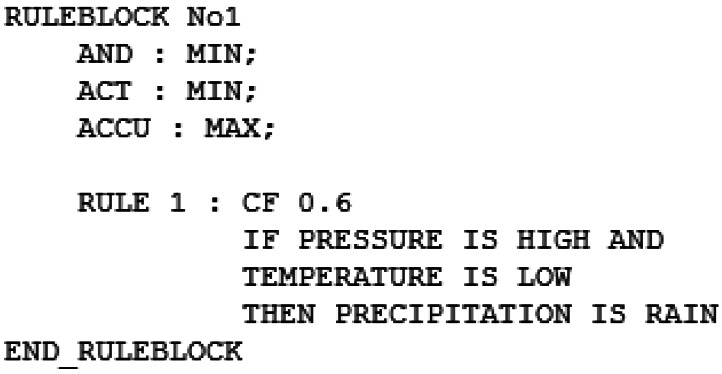
An example of an uncertain fuzzy rule.

The second type of uncertainty takes place when the outcome of a rule is not fixed, for example “if the barometric pressure is high and the temperature is low there is a 60% chance of rain”. To model this aspect of uncertainty we have modified the grammar of the FCL language. An example of the modified rules can be seen in Figure 7.

Uncertainty and fuzziness can appear in the same rule and influence each other. To tackle this problem we have implemented the inference model described in [29]. This model contemplates three different situations depending on the nature of the antecedent and consequent of the rule and the matching fact: CRISP Simple Rule where both antecedent and matching fact are crisp values, FUZZY_CRISP Simple Rule where both the antecedent and matching fact are fuzzy and the consequent is crisp and finally the FUZZY_FUZZY Simple rule where all three are fuzzy.

In the case of the CRISP Simple Rule the certainty factor of the consequent is calculated using the following formula:

(2) $$CF_{c}=CF_{r}\times CF_{f}$$

where:

- *CF_c_*: the certainty factor of the consequent.
- *CF_r_*: the certainty factor of the rule
- CF_f_: the certainty factor of the fact

In the case of the FUZZY_CRISP Simple Rule, the certainty factor of the consequent is calculated using the following formula:

(3) $$CF_{c}=CF_{r}\times CF_{f}\times S$$

where S is the measurement of similarity between both fuzzy sets and is calculated using the following formula:

(4) $$\begin{array}{cc} S=P\left(F_{\alpha }|F_{\alpha }^{\prime }\right) & if\;\text{N}\left(F_{\alpha }|F_{\alpha }^{\prime }\right)>0.5\\ S=\left(N\left(F_{\alpha }|F_{\alpha }^{\prime }\right)+0.5\right)\times P\left(F_{\alpha }|F_{\alpha }^{\prime }\right) & \text{otherwise} \end{array}$$

where:

(5) $$\begin{array}{cc} P\left(F_{\alpha }|F_{\alpha }^{\prime }\right)=\text{max}\left(\text{min}\left(\mu_{F_{\alpha }}\left(u\right),\mu_{F_{\alpha }^{\prime }}\left(u\right)\right)\right), & \forall u\in U \end{array}$$

and:

(6) $$N\left(F_{\alpha }|F_{\alpha }^{\prime }\right)=1-P\left(\bar{F_{\alpha }}|F_{\alpha }^{\prime }\right)$$

Finally in the case of the FUZZY_FUZZY Simple Rule the certainty factor of the consequent is calculated using the same formula as the CRISP Simple RULE. Currently we do not support this type of combined reasoning for complex rules that involve multiple clauses in their antecedent.

## 5. Use Cases

In order to illustrate the importance of modeling ambiguity and having a data fusion process in context management systems we will describe several smart environment scenarios. Each scenario will help us to show different aspects closely related to our systems: modeling the certainty of the captured measures and the rules, modeling the subjectivity in the perceptions of the users and the importance of creating a global picture for each space.

### 5.1. Scenario 1: Temperature Control in a Laboratory

In this first scenario we will explain the importance of evaluating the certainty factor of the context data and the importance of the data fusion process. In this scenario we want to maintain the temperature of our laboratory at 23 degrees. To do this we will use four thermometers (see Table 2) located inside the lab to measure the temperature. The laboratory has a radiator to regulate the temperature and to simplify the example we will assume that the only operation it allows is to switch it on and off. The low certainty factor in “Thermometer 4” is due to the fact this thermometer is broken. For this example we assume that each thermometer has a self-diagnostic mechanism that constantly evaluates its status. The self-diagnostic details fall outside the scope of this example.

The scenario will have three versions (see Table 3). The first one will use a semantic model without uncertainty and vagueness information and without any data fusion process. The second one will include a rather simple average value calculation as the data fusion mechanism. Finally, the third one will be the process previously described. In each scenario we will describe the sensors and actuators that take part, their values and CF and the rules that model the behavior of the system. To make the examples easier to understand the rules will be written in pseudocode using simple triple-like expressions.

In the first version, with no ambiguity modeling or data fusion (see Table 3), the rules would be the following: to switch on the radiator:

thermoX type thermometer,
thermoX location LaboratoryA
thermoX hasValue temperatureX
temperatureX < 21
>
radiatorX type radiator
radiatorX location LaboratoryA
radiator status ON

And to switch it off:

thermoX type thermometer,
thermoX location LaboratoryA
thermoX hasValue temperatureX
temperatureX > 24
>
radiatorX type radiator
radiatorX location LaboratoryA
radiator status OFF

There are several problems with this approach. First the system is not able to take into account the low reliability of the thermometer T4, using its temperature measure as input for the reasoning engine. This will result in incorrect behavior when that measure is processed, prompting the system to switch on the radiator to increase the room temperature. Secondly, each temperature measurement is processed individually, resulting in unreliable results that depend on the order of this processing. This version the system has no means of knowing the global measure for the laboratory and must process each measure individually, resulting in contradictory actions in this case.

In the second version (see Table 3) a simple data fusion mechanism will be used. This example will help us illustrate the need of a sound and adaptable data fusion mechanism. The rules that control the behavior will be the following: to switch on the radiator:

LaboratoryA hasGlobalTemperature temperatureX
temperatureX < 21
>
radiatorX type radiator
radiatorX location LaboratoryA
radiator status ON

And to switch it off:

LaboratoryA hasGlobalTemperature temperatureX
temperatureX > 25
>
radiatorX type radiator
radiatorX location LaboratoryA
radiator status OFF

In this case the system has a unified vision of the temperature in the room and is able to take an unique action to adjust the radiator, but still does not take into account the differences in the certainty factors of the sensors. As a result the global temperature in the laboratory is 20.25 °C and the radiator will be turned on.

Finally in the third version of the scenario we use the system described previously, processing the ambiguity of the context data and applying a richer data fusion process. In this case we use the combination strategy with a cautious behavior for the data fusion process (see Section 4.3). The rules are almost identical to the second version, but include an important change—now they state a minimum CF for body of the rule to be launched. The rules that control the behavior will be the following: to switch on the radiator:

LaboratoryA hasGlobalTemperature temperatureX
temperatureX < 21 WITH CF 0.8
>
radiatorX type radiator
radiatorX location LaboratoryA
radiator status ON

And to switch it off:

LaboratoryA hasGlobalTemperature temperatureX
temperatureX > 25 WITH CF 0.8
>
radiatorX type radiator
radiatorX location LaboratoryA
radiator status OFF

According to the selected configuration the global temperature value will be:

(7) $$m_{\text{global}}=\frac{\left(25*0.85+23*0.95+24*0.9+11*0.4\right)}{0.85+0.95+0.9+0.4}≅22.3$$

And the global certainty factor:

(8) $$CF_{\text{global}}=\frac{0.85+0.95+0.9+0.4}{4}=0.775$$

The certainty factor of the global measure will be too low to launch the rule (we set a minimum CF of 0.8 for the temperature in the rules), but we can tune even more the configuration and establish a CF threshold for the measures in the data fusion process. As we explained in Section 4.3 using the threshold the measurements with a CF below it are automatically discarded. Setting the threshold for the data fusion problem at 0.8 the measurements given by the broken thermometer are discarded, obtaining the following results:

(9) $$m_{\text{global}}=\frac{25*0.85+23*0.95+24*0.9}{0.85+0.95+0.95}≅23.96$$

And the global certainty factor:

(10) $$CF_{\text{global}}=\frac{0.85+0.95+0.9}{3}=0.9$$

Taking into account the certainty factor of the context data and applying a data fusion process the system can ascertain much more reliably the real state of the laboratory, providing a better picture of its current situation. As a result the system knows that the temperature value is between the acceptable limits and that no action must be taken.

As can be seen on Table 4 the results between different versions vary significantly. The results in the first version are completely unreliable, as the final state depends on the order that the rules are evaluated (this is problematic because several semantic reasoning engines do not guarantee the order in which matching rules will fire [30]). The second version uses the measurement from the broken thermometer to compute the global measure, inducing an error in the calculation that results in the radiator been switched on. Finally, the last version takes into account the certainty factor, resulting in a more reliable system.

### 5.2. Scenario 2: Occupation Data Inferred from the Status of the Lights

This scenario will help us illustrate the necessity of modeling another aspect of the uncertainty. In the previous scenario we have explained the importance of assessing the uncertainty in the taken measurements; this one will be centered in the uncertainty of the rule. In this scenario we want to infer room occupation data based on the status of the lights. We will have two versions of this scenario with and without uncertainty.

The first version is quite straightforward, without taking into account the outcome uncertainty. The rules that model the behavior will be:

lightX type light,
lightX location LaboratoryA
lightX hasValue On
>
LaboratoryA hasStatus peopleInside

In this case, we assume that if the lights of the laboratory are ON, there is someone inside. But we do not take into account situations where the users get out leaving the lights on. Using the rule uncertainty we could model these situations. Assuming that after studying the habits of the users we have inferred that 90% of the time, when a light is on there is someone in the laboratory, then the resulting rule would be:

RULE CF 0.9
lightX type light,
lightX location LaboratoryA
lightX hasValue On
>
LaboratoryA hasStatus peopleInside

Using the rule uncertainty we can better model the contextual information (in this case the information obtained after processing the raw data from the measurements). The result is a more reliable model of the smart environment that ascertains better its real status.

### 5.3. Scenario 3: User Preferences for Temperature

This final scenario will help us explain the importance of taking into account the human factor when modeling the context data. In our case we use vagueness to model the user perceptions and represent the vagueness using fuzzy sets. This scenario will be similar to the first one, but the temperature limits will be expressed using fuzzy facts. To show how the uncertainty and the vagueness interact it will be a FUZZY-CRISP simple rule with fuzzy uncertain input facts and a crisp output:

- Input data: Fuzzified room temperature with an associated certainty factor (0.95 in this example). We assume that the fuzzification process is done accordingly to the previously captured user perceptions.
- Output data: Crisp temperature value for the air conditioning system with a certainty factor determined using the third formula explained in Section 4.1.
- Rule: FUZZY-CRISP simple rule with an associated certainty factor.

The used rule will be:

RULE CF 1
LaboratoryA hasGlobalTemperature temperatureX
temperatureX HOT
>
airCoditioningX type AirConditioning
airCoditioningX location LaboratoryA
airCoditioningX temperature 22

**Figure 8.**
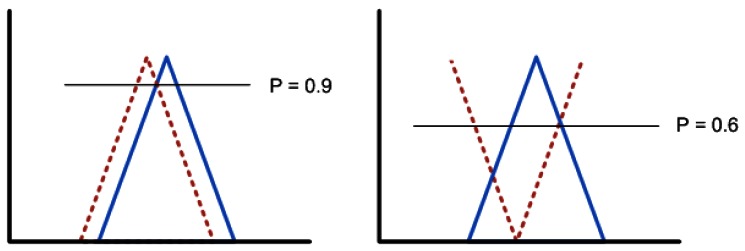
Possibility calculation. The straight line represents the expected fuzzy fact and the dotted line the provided fuzzy fact.

The first step will be to calculate the value of $\text{P}({\text{F}}_{\alpha }|{\text{F}}_{\alpha }^{\prime })$ (see Figure 8 for a graphical explanation) and with it the measure of necessity (N) [see formula (6)]:

(11) $$N\left(F_{\alpha }|F_{\alpha }^{\prime }\right)=1-0.6=0.4$$

N is lower than 0.5, so the similarity (S) measure will be:

(12) $$S=\left(N\left(F_{\alpha }|F_{\alpha }^{\prime }\right)+0.5\right)\times P\left(F_{\alpha }|F_{\alpha }^{\prime }\right)=\left(0.4+0.5\right)\times 0.9=0.81$$

With the similarity we can calculate the certainty factor of the outcome following formula (3):

(13) $$CF_{c}=CF_{r}\times CF_{f}\times S=1\times 0.95\times 0.81=0.76$$

Using fuzzy sets we can model the vagueness present in the context data. This allows us to model user perceptions and preferences (e.g., “I like the water hot”, “Turn on the air conditioning system when the room is hot”) without the loss of expressiveness that we will have using only crisp values.

## 6. Discussion and Future Work

In this paper we have presented an ontology that models the ambiguity of smart environments and a flexible semantic context management system. Our context management system is able to process the uncertainty and vagueness of the contextual information. We have also described a data fusion mechanism applied in the case that multiple data sources for the same measurement exist in one room. In the previous section we have explained how the inclusion of these three elements (uncertainty, vagueness and data fusion) in context management systems provides several advantages:

- Taking into account uncertainty and vagueness of the contextual information provides a more detailed picture of the current state of the user environment. This leads to more informed decisions of the Smart Environments.
- Uncertainty allows us to ascertain the quality of the data, discarding those measurements below a certain threshold. The result of the more robust data model is the improvement of the system reliability.
- Vagueness allows us to model user perceptions. This allows us to react better to their requirements.
- The necessity of a data fusion process in context managing system can be clearly seem in those scenarios where the environment contains a large number of sensors. To tackle this problem we try to provide a unified picture of the environment to avoid conflicting behaviors.

The created system has also some constraints. The most important one is the extra knowledge required from the system-deployers to model both aspects of the ambiguity (uncertainty and vagueness) when designing the domain specific applications. A broader knowledge of the domain is necessary in order to correctly create the rules according to the different certainty factors. System-deployers are also required to model the fuzzy membership functions for the different measurements, but this same problem arises when a traditional fuzzy inference engine is used. Finally is necessary to ascertain the certainty factor of each sensor to be able to provide the certainty of the taken measures. If data about the sensor capabilities is not provided by the vendor, system-deployers would have to perform a testing phase prior to the system deployment to determine these capabilities. Despite of these drawbacks the proposed context managing system will provide system-deployers with a more truthful view of the situation of the modeled smart environment. The extra work taken when modeling the environment will lead to more informed decisions. This will help to avoid the unwanted behavior discussed in the previous section, like performing actions using unreliable context information given by broken sensors or processing multiple measures of the same location. The result will be a more reliable context management process.

As discussed in the related work section, our solution differs from previous ambiguity modeling approaches in that it combines both aspects of the ambiguity in one solution that also provides a semantic model of the environment. As a result we are able to model a broader range of situations and interactions, providing richer context information. It also differs from mediation-based approaches like [31] in that our system does not require any feedback from the user to identify and ascertain the uncertainty. Non-expert users will find difficult to evaluate the certainty of the raw data of the sensors. Instead of using a feedback process to appraise the certainty factor of more abstract context information, we have implemented a data fusion process that calculates the combined uncertainty of the measurements of a specific location and a reasoning process that infers the certainty factor of the data created from the combination of raw measurements. In any case, it would be interesting to add some kind of feedback from system-deployers or an automatic method to assess the validity of the calculated certainty factor in order to allow the system to learn and infer the correct context data.

As future work, first we would like to create a mechanism that automatically assesses the certainty factor of a sensor comparing its data with the one provided by other sensors. This will allow us to identify and discard malfunctioning sensors. We would also like to include a feedback based learning mechanism that fine-tunes over the time the certainty and vagueness data of the modeled intelligent environment.

Secondly we would like to develop an ecosystem of reasoners to distribute the inference process. We have already created an initial version of this inference sharing process [32] basing our approach on an agent based peer-to-peer architecture that divides the reasoning problem into smaller inference units according to the interests stated by each of the context consumers. Inference is no longer performed by a central reasoning engine, making it more computationally affordable and allowing us to combine less powerful devices to obtain a rich and expressive inference. We expect to achieve several objectives with this approach to context reasoning:

1. Attain the temporal decoupling of the different inference units, allowing the inference to be done concurrently in various reasoning engines.
2. Attain the spatial decoupling of the inference process, increasing the general robustness of the system and making it more fault-tolerant.
3. Reduce the number of triples and rules that each reasoning engine has to manage, which will allow us to use more computationally constrained devices to carry out the inference process.

Combining these characteristics with those already explained in this paper will result in a distributed, robust and fault-tolerant context management process that is able to process the ambiguity information and can be deployed in more computationally limited devices.

## Figures and Tables

**Table 1.** Example of the data fusion.

	**Value**	**CF**	**Global Value**	**CF Severe**	**CF Indulgent**	**CF Cautious**
**M1**	18	0.7	19.9	0.6	0.8	0.7
**M2**	22	0.7				
**M3**	16	0.6				
**M4**	20	0.8				

**Table 2.** Devices present in scenario 1.

**Device**	**Type**	**CF**	**Value**

Thermometer 1	Sensor	0.8	25
Thermometer 2	Sensor	0.95	23
Thermometer 3	Sensor	0.9	22
Thermometer 4	Sensor	0.6	11
Radiator	Actuator	NA	NA

**Table 3.** Configuration of each version of the scenarios.

	**Uncertainty**	**Vagueness**	**Data fusion**
**Version 1**	No	No	No
**Version 2**	No	No	Yes
**Version 3**	Yes	Yes	Yes

**Table 4.** Results of each version of the first scenario.

	**Version 1**	**Version 2**	**Version 3**
**Result**	The radiator controlling rules are fired two times (only two of the four values meet the conditions). The final state of the radiator will depend on the order in which the rules are evaluated	The computed global measure will be 20.25 °C. The radiator will be turned on.	The computed global measure will be 23.96 °C. No action will be taken.
